# Communication Anxiety and Bicultural Engagement in Relation to Parenting Efficacy Among Chinese Marriage Migrant Women in Korea: The Mediating Role of Parenting Stress

**DOI:** 10.3390/bs16091677

**Published:** 2026-09-17

**Authors:** Linge Guan, Yujin Jang

**Affiliations:** Early Childhood Education, College of Social Science, Gachon University, Seongnam-si 13120, Republic of Korea; glgbhh@gachon.ac.kr

**Keywords:** communication anxiety, bicultural engagement, parenting stress, parenting efficacy, Chinese marriage migrant women

## Abstract

This study examined the relationships among communication anxiety, bicultural engagement, parenting stress, and parenting efficacy among Chinese marriage migrant women living in Korea, with particular attention to the mediating role of parenting stress. Using cross-sectional survey data, the study tested a structural model linking communication anxiety and acculturation to parenting efficacy through parenting stress. The results showed that higher communication anxiety was associated with higher parenting stress, whereas higher bicultural engagement was associated with lower parenting stress. Parenting stress, in turn, was negatively associated with parenting efficacy. The direct relationship between communication anxiety and parenting efficacy was not significant, while the indirect relationship through parenting stress was significant. Bicultural engagement showed a positive indirect relationship with parenting efficacy through lower parenting stress, although its direct relationship with parenting efficacy was in the opposite direction. These findings suggest that communication anxiety and bicultural engagement represent distinct aspects of migration-related experience and are differently associated with parenting. They also highlight parenting stress as an important mechanism linking migration-related experiences with mothers’ perceptions of parenting competence. The findings underscore the need for support programs that address communication-related anxiety, cultural adjustment, and parenting stress among Chinese marriage migrant women in Korea.

## 1. Introduction

Korea has historically emphasized ethnic homogeneity, a tendency that has shaped both education and public understandings of national identity. Since the early 2000s, the rapid increase in international marriage and migration has led the Korean government to expand support for multicultural families, particularly marriage migrant women. Although these policies provided important institutional support, early integration efforts placed considerable emphasis on Korean-language acquisition, familiarity with Korean customs, and adjustment to established family and social norms. Accordingly, Korea’s multicultural policies have long been characterized as containing assimilation-oriented elements (H. M. Kim, 2007; Zmire, 2022). The Multicultural Families Support Act, enacted in 2008, institutionalized support for the settlement and family life of marriage migrants, linking their integration into Korean society closely with successful functioning within the family. For marriage migrant women, acculturation therefore unfolds across both family and community contexts.

According to statistics from the Korean Ministry of Justice at the end of June 2026, approximately 190,000 foreign residents in Korea resided under marriage migrant (F-6) status, of whom more than 60,000 were Chinese nationals, including ethnic Koreans from China, representing the largest national group (Ministry of Justice, 2026). Women accounted for more than 80% of these residents. When naturalized marriage migrants are also considered, Chinese marriage migrant women constitute a substantial segment of Korea’s multicultural female population. Attention to their settlement and parenting experiences is therefore warranted. They must navigate an unfamiliar linguistic and institutional environment while negotiating culturally specific expectations regarding family roles and parenting in Korean society (S. Lee & Lee, 2023).

Research on Chinese marriage migrant women in Korea has shown that linguistic and cultural barriers can restrict access to information, services, and social resources (J. Kim et al., 2022; Ko & Kim, 2024; S. Lee & Lee, 2022). Such barriers may be particularly consequential in parenting, which requires frequent communication with childcare and educational institutions, healthcare providers, and other parents. Importantly, these difficulties cannot be explained by Korean-language proficiency alone. Even women with adequate language skills may experience communication anxiety because of concerns about misunderstanding, making mistakes, or being negatively evaluated when speaking Korean (Ko & Kim, 2024). Communication anxiety may therefore affect mothers’ confidence in obtaining information, expressing concerns about their children, and participating in educational and community settings.

These challenges are not unique to the Korean context. International research suggests that migration-related stressors may become intertwined with parenting experiences among immigrant families. Among low-income immigrant parents of preschool-aged children in the United States, higher perceived stress was associated with lower parenting self-efficacy, although relational empowerment buffered this association (Park et al., 2025). Discrimination may constitute an additional contextual burden. For example, research with Latino immigrant parents in the United States found that higher levels of perceived discrimination were associated with less favorable parenting patterns, including lower monitoring and consistent discipline and greater harsh discipline (Ayón & García, 2019). Together, these findings suggest that linguistic and cultural adaptation takes place within broader social conditions that may either intensify or alleviate the demands of parenting. Although discrimination was not directly assessed in the present study, it represents an important contextual factor when considering the parenting experiences of marriage migrant women.

Acculturation represents another important dimension of these experiences. From a bidimensional perspective, adaptation does not require immigrants to abandon their heritage culture in favor of the host culture; orientations toward Chinese and Korean cultures can coexist (Berry, 1997). In the present study, acculturation was conceptualized as bicultural engagement, reflecting orientations toward both Chinese and Korean cultures. This perspective is particularly relevant in Korea, where earlier assimilation-oriented expectations emphasized adjustment to the Korean language and cultural norms (Zmire, 2022). The closest precedent to the present study is S. Jang and Lee (2026), who showed that acculturative stress among immigrant mothers in Korea was associated with less adaptive parenting processes and, indirectly, with negative developmental outcomes for children. The present study extends this line of research by distinguishing communication anxiety from bicultural engagement and by examining parenting stress as a mechanism linking these distinct migration-related experiences to mothers’ parenting efficacy. These findings suggest that the linguistic and cultural challenges experienced by marriage migrant women may be associated with parenting both directly and through psychological burdens related to the parental role. Parenting stress may therefore serve as a key mechanism linking communication anxiety and acculturation to parenting efficacy. Repeated difficulties in communication, obtaining information, and managing unfamiliar parenting expectations may increase parenting stress and, in turn, weaken mothers’ confidence in their parenting abilities.

Parenting stress is particularly important because it reflects more than the everyday demands of caring for a child. It arises from the interplay among parental resources, perceptions of the child, and the quality of the parent–child relationship, and may shape how parents evaluate their own competence (H. J. Kim & Lim, 2022). Korean family research has similarly emphasized that parenting and family functioning are shaped not only by individual characteristics but also by broader relational and social contexts (Ahn, 2024; J. Lee et al., 2023). Taken together, these studies show that parents’ psychological functioning is closely tied to the social and relational contexts in which parenting occurs.

These issues may be particularly salient for marriage migrant mothers. The demands of raising young children are accompanied by the additional task of navigating unfamiliar institutions, social norms, and interpersonal expectations. The present study therefore focused on mothers of children aged 2–5 years, a period in which parents remain closely involved in everyday caregiving while also increasingly interacting with childcare, educational, and healthcare settings on behalf of their children. In this context, parenting efficacy may be closely tied to mothers’ confidence in managing both direct caregiving demands and communication with institutions and other adults involved in their children’s lives. For non-migrant mothers, communicating with teachers, seeking health information, attending parent meetings, and interpreting institutional guidance may be routine parenting tasks. When these tasks must be carried out in a second language and an unfamiliar cultural context, however, they may require substantial emotional and cognitive effort. Previous research on Chinese marriage migrant women in Korea has shown that linguistic and cultural accessibility can influence their ability to use community-based services effectively (J. Kim et al., 2022). From this perspective, parenting stress among marriage migrant women reflects the multiple demands that arise at the intersection of migration and parenthood.

Parenting efficacy refers to parents’ beliefs that they can successfully perform parenting tasks and positively influence their children (Back & Jang, 2023; Che et al., 2026). Parents who perceive themselves as competent are generally more likely to view difficulties as manageable and to persist in demanding parenting situations (Y.-J. Jang & Yin, 2023). In contrast, repeated experiences of stress or failure may weaken confidence in the parental role. Previous studies have consistently identified self-efficacy and family support as important resources associated with parental adaptation and well-being. The relationship between parenting stress and parenting efficacy may therefore be especially important for Chinese marriage migrant mothers who have limited support from their family of origin or social networks.

Communication anxiety may make everyday child-related interactions more stressful, while difficulties in adjusting to Korean cultural expectations may increase uncertainty about appropriate parenting (Boruszak-Kiziukiewicz & Kmita, 2020). These experiences do not necessarily reduce parenting efficacy directly. Rather, their associations with parenting efficacy may operate through parenting stress. As everyday parenting situations become more demanding and less predictable, mothers may become less confident in their ability to manage them effectively (Song et al., 2022). This possibility is consistent with a social-contextual view of parenting, in which parental confidence develops through repeated interactions with family members, institutions, and the broader social environment. The proposed ordering reflects a stress-process interpretation in which communication anxiety and bicultural engagement are treated as broader migration-related experiences that may shape mothers’ stress within the parenting role. Parenting stress, in turn, represents a more proximal parenting-specific experience that may be associated with mothers’ evaluations of their parenting competence. This ordering is theoretically plausible, although the cross-sectional design of the present study does not exclude reciprocal or reverse relationships.

At the same time, communication anxiety and acculturation should not be treated as interchangeable aspects of migration. Communication anxiety concerns apprehension in actual interpersonal situations, whereas acculturation refers to the broader process of cultural orientation and participation in the receiving society (Y. Jang et al., 2007; van der Zee & van Oudenhoven, 2022). A mother may still experience anxiety in specific communication situations while being relatively well acculturated to Korean society. Conversely, she may maintain a strong orientation toward Chinese culture while communicating confidently in Korean. Examining these factors together may therefore provide a more differentiated understanding of how migration-related experiences become relevant to parenting.

Despite growing research on marriage migration in Korea, relatively few studies have examined communication anxiety and acculturation together with parenting efficacy, with particular attention to the mediating role of parenting stress. Previous studies have more often focused on Korean-language proficiency, general acculturative stress, or social adaptation as independent predictors of psychological adjustment (J. S. Kim, 2018; Boruszak-Kiziukiewicz & Kmita, 2020). Li and An (2023) provide a particularly relevant point of comparison. In their study of Chinese, Vietnamese, and Japanese marriage migrant women, communication problems and parenting efficacy were identified as important correlates of parenting stress, with the pattern differing across national groups. The present study extends this work in several ways. Rather than treating communication difficulty as one component of general acculturative stress, we distinguish communication anxiety from bicultural engagement as separate migration-related experiences. In addition, parenting stress is examined as an intervening mechanism linking these experiences with parenting efficacy rather than primarily as an outcome. Less attention has been given to the possibility that communication-related anxiety and acculturation may be differently associated with mothers’ perceptions of parenting competence through parenting stress. Addressing this gap is important in the Korean context, where marriage migrant women often negotiate linguistic and cultural adjustment while also assuming substantial responsibility for the care and education of young children.

Accordingly, this study examined the relationships of communication anxiety and acculturation with parenting efficacy among Chinese marriage migrant women living in Korea, with particular attention to the mediating role of parenting stress. By considering linguistic, cultural, and parenting-related experiences within a single structural model, the study aimed to clarify how migration-related challenges are associated with mothers’ beliefs about their parenting competence. Based on the preceding theoretical rationale, the following hypotheses were formulated:

**H1.**  *Higher communication anxiety will be associated with higher parenting stress.*

**H2.**  *Greater bicultural engagement will be associated with lower parenting stress.*

**H3.**  *Higher parenting stress will be associated with lower parenting efficacy.*

**H4.**  *Parenting stress will mediate the association between communication anxiety and parenting efficacy.*

**H5.**  *Parenting stress will mediate the association between bicultural engagement and parenting efficacy.*

## 2. Methods

### 2.1. Participants

A total of 158 Chinese marriage-immigrant women residing in Seoul, Gyeonggi Province, and Incheon and raising children aged 2 to 5 years participated in the study. In the present study, Chinese marriage-immigrant women were defined as women of Chinese origin who migrated to Korea through marriage to a Korean national, initially obtained F-6 (Marriage Migrant) residence status, and subsequently established long-term residence and family life in Korea. Women who had initially entered or resided in Korea under F-6 status but later acquired Korean citizenship were also included. Thus, eligibility was determined on the basis of participants’ marriage-migration background rather than their current nationality alone.

Data were collected through both online and offline surveys. For the online survey, 110 questionnaires were distributed and returned via Google Forms. An additional 50 paper-and-pencil questionnaires were collected through multicultural family centers and community-based gatherings. Of the 160 questionnaires returned, two paper questionnaires were excluded because of incomplete or careless responses.

Among the participants, 67 (42.4%) had a 5-year-old child, representing the largest child-age group. Most participants had one child (n = 99, 62.7%). Regarding maternal age, 103 participants (65.2%) were in their 30s. In terms of educational attainment, 54 participants (34.2%) had completed a university degree, and 45 (28.5%) were enrolled in or had completed graduate-level education. The majority had lived in Korea for 7 years or longer (n = 100, 63.2%), followed by 3–6 years (n = 37, 23.4%) and less than 3 years (n = 21, 13.2%). With respect to Korean-language learning, 97 participants (61.3%) reported having studied Korean for 1–3 years, 32 (20.2%) for 3–6 years, and 29 (18.3%) for 7 years or longer. Self-rated Korean proficiency was most commonly reported as intermediate (n = 81, 51.2%), followed by advanced (n = 47, 29.7%) and beginner level (n = 30, 18.9%). Regarding language use at home, 57 participants (36.1%) reported using both Korean and Chinese, 56 (35.4%) primarily used Korean, and 45 (28.5%) primarily used Chinese. In terms of national and ethnic background, 121 participants (76.5%) were of Han Chinese or other Chinese ethnic backgrounds, 20 (12.7%) were ethnic Koreans from China (Joseonjok), and 17 (10.7%) had acquired Korean nationality. Participants were recruited through convenience sampling via multicultural family support centers and community-based gatherings; consequently, the sample was not intended to be nationally representative of Chinese marriage migrant women in Korea.

### 2.2. Procedure

The study was conducted in two stages: a pilot study followed by the main survey. The pilot study involved four Chinese marriage-immigrant women residing in Korea and was conducted using paper-and-pencil questionnaires. Its primary purpose was to assess the comprehensibility of the items and the appropriateness of the response format. The study protocol was reviewed and approved by the Institutional Review Board of Gachon University. The pilot study identified several expressions that could potentially cause confusion among participants. For example, differences between Korean and Chinese age-counting conventions created ambiguity when reporting children’s ages. Accordingly, the child’s birth year was provided alongside each age category in the main survey (e.g., age 3 [born in 2021]) to improve response accuracy. In addition, the wording of several items was revised and refined in consultation with an expert in early childhood education to enhance their conceptual and linguistic appropriateness.

Data for the main study were collected using both online and offline methods. Before data collection, informed consent was obtained from all participants. The online survey was administered through Google Forms, whereas paper-and-pencil questionnaires were distributed and collected through gatherings organized by multicultural family support centers. Prior to participation, respondents were provided with information regarding the purpose of the study, their rights as research participants, and the protection of personal information. Participation was entirely voluntary and based on informed consent. Completion of the questionnaire required approximately 15 min.

To accommodate participants’ varying levels of Korean-language proficiency and enhance response accuracy, the questionnaire was provided in both Korean and Chinese. Translation was conducted following the back-translation procedure proposed by Brislin (1970). The Korean questionnaire was first translated into Chinese by the researcher. The translated version was then reviewed by two Chinese graduate students majoring in early childhood education in Korea, one enrolled in a master’s program and the other in a doctoral program, who examined the wording and suggested revisions. The revised Chinese version was subsequently back-translated into Korean by a Chinese doctoral student majoring in Korean language and literature to assess semantic equivalence between the two language versions. Finally, the back-translated version was independently cross-checked by a Korean reviewer who had resided in China for more than five years and had intermediate-to-advanced Chinese proficiency, with particular attention to linguistic naturalness and accuracy. Through this multistep translation and review process, efforts were made to maximize the linguistic equivalence and cultural appropriateness of the questionnaire across the Korean and Chinese versions.

### 2.3. Measures

#### 2.3.1. Communication Anxiety

Communication anxiety was assessed using selected items from the Foreign Language Classroom Anxiety Scale (FLCAS) developed by Horwitz et al. (1986). The original FLCAS consists of 33 items rated on a five-point Likert scale and was designed to assess anxiety specifically associated with foreign-language learning and use. Because the present study focused on anxiety arising during everyday communication in Korean rather than anxiety specific to language-learning situations, items referring primarily to tests, classroom performance, or other classroom-specific experiences were not retained. Eleven items that reflected communication apprehension and concerns about misunderstanding, making mistakes, or being negatively evaluated during interpersonal communication were selected. The retained items were those that could be meaningfully adapted to everyday communication without substantially altering the underlying anxiety construct. Participants responded to each item on a five-point Likert scale, with higher scores indicating greater anxiety when communicating in Korean in everyday situations. In the present study, the 11-item communication anxiety measure demonstrated high internal consistency (Cronbach’s α = 0.92).

For the structural equation model, the 11 communication anxiety items were grouped into three parcels using an item-to-construct balancing approach. Items were first ordered according to their standardized factor loadings and then distributed across the three parcels in a balanced sequence so that relatively high- and low-loading items were represented across parcels. The resulting parcels contained four, four, and three items, respectively.

#### 2.3.2. Bicultural Engagement

Bicultural engagement was assessed using the Vancouver Index of Acculturation (VIA) developed by Ryder et al. (2000). The VIA was originally developed within a bidimensional framework of acculturation and assesses orientations toward heritage and mainstream cultures as conceptually distinct but potentially coexisting dimensions. In the present study, bicultural engagement was conceptualized as the extent to which Chinese marriage migrant women maintained orientation toward their heritage culture while simultaneously engaging with the cultural context of Korean society. Thus, Chinese and Korean cultural orientations were regarded as complementary dimensions of a broader bicultural engagement construct rather than as opposite ends of a single continuum. This specification does not assume that Chinese and Korean cultural orientations are interchangeable or represent a single undifferentiated dimension. Rather, bicultural engagement was conceptualized as a broader construct reflecting concurrent involvement in both cultural contexts, while the Korean- and Chinese-orientation components were retained separately as distinct indicators in the measurement model. The modest positive correlation between the two orientations (r = 0.33) is consistent with the VIA’s bidimensional premise that engagement with one cultural context does not preclude engagement with the other.

Because all participants were Chinese marriage migrant women residing in Korea, references to the heritage culture were specified as Chinese culture, whereas references to mainstream North American culture were adapted to Korean culture. Two items concerning willingness to marry a person from one’s heritage or mainstream culture were excluded because they were not applicable to the present sample of married women. The final measure therefore consisted of 18 items, including nine assessing Chinese Cultural Orientation and nine assessing Korean Cultural Orientation. The original VIA uses a nine-point Likert-type response scale; however, a five-point scale was adopted in the present study to provide a consistent response format across the questionnaire. Higher scores on each dimension indicated stronger orientation toward the respective culture. Cronbach’s α was 0.83 for Chinese Cultural Orientation and 0.83 for Korean Cultural Orientation. For the structural equation model, the nine items assessing each cultural orientation were grouped into two parcels using an item-to-construct balancing approach based on factor loadings. This resulted in two indicators each for Korean Cultural Orientation and Chinese Cultural Orientation.

#### 2.3.3. Parenting Self-Efficacy

Parenting self-efficacy was assessed using the Korean version of the Échelle Globale du Sentiment de Compétence Parentale (K-EGSCP), originally developed by Meunier and Roskam (2009) and subsequently translated and validated for Korean parents by Sung and Baek (2011). The original EGSCP was designed to assess parents’ domain-specific self-efficacy beliefs as well as related parental cognitive constructs. Sung and Baek (2011) adapted the original scale for Korean parents and established a seven-factor structure through exploratory and confirmatory factor analyses.

For the present study, only the 22 items assessing the five domains of parenting self-efficacy were used, excluding items assessing the related cognitive constructs of parental responsibility and parental control of outcomes. The five domains were Discipline (5 items), Play (5 items), Nurturance (5 items), Instrumental Care (4 items), and Teaching (3 items). Discipline assesses parents’ perceived efficacy in guiding their children’s behavior and promoting compliance with rules. Play reflects parents’ confidence in engaging and interacting effectively with their children through play. Nurturance assesses perceived efficacy in providing affection, emotional responsiveness, and support. Instrumental Care reflects parents’ confidence in organizing and managing their children’s daily routines and practical caregiving needs. Teaching assesses parents’ perceived ability to provide developmentally appropriate explanations, guidance, and learning experiences.

Items were rated on a five-point Likert scale, with higher scores indicating greater parenting self-efficacy. In the present study, Cronbach’s α was 0.85 for Discipline, 0.86 for Play, 0.79 for Nurturance, 0.78 for Instrumental Care, and 0.80 for Teaching. Cronbach’s α for the overall parenting self-efficacy scale was 0.92.

#### 2.3.4. Parenting Stress

Parenting stress was assessed using the Korean version of the Parenting Stress Index–Short Form (K-PSI-SF), originally developed by Abidin (1995) and validated for Korean parents by K. S. Lee et al. (2008). The PSI-SF is designed to assess stress experienced within the parent–child relationship and consists of 36 items across three subscales: Parental Distress (PD), Parent–Child Dysfunctional Interaction (P-CDI), and Difficult Child (DC), with 12 items in each subscale. The Parental Distress subscale assesses parents’ personal distress associated with the parenting role, including perceived restrictions, emotional burden, and difficulties in fulfilling parental responsibilities. The Parent–Child Dysfunctional Interaction subscale assesses parents’ perceptions that interactions with their child do not meet their expectations and reflects dissatisfaction with the quality of the parent–child relationship. The Difficult Child subscale assesses stress associated with children’s temperament, behavioral characteristics, and the perceived difficulty of managing and caring for the child.

Responses were scored on a five-point scale in accordance with the K-PSI-SF scoring procedure, with higher scores indicating greater levels of parenting stress. In the present study, Cronbach’s α was 0.71 for Parental Distress, 0.82 for Parent–Child Dysfunctional Interaction, and 0.87 for Difficult Child. Cronbach’s α for the total scale was 0.91.

### 2.4. Data Analysis

Analyses were conducted using IBM SPSS Statistics 27.0 and AMOS 20. Parameters were estimated using maximum likelihood estimation. Prior to the SEM analyses, the data were screened for missing values, univariate and multivariate normality, outliers, and multicollinearity. Descriptive statistics and Pearson’s correlation coefficients were calculated to examine the distributions and bivariate relationships among the study variables. Confirmatory factor analysis (CFA) was conducted to evaluate the measurement model, followed by structural equation modeling (SEM) to examine the relationships of communication anxiety and bicultural engagement with parenting efficacy and the mediating role of parenting stress. Bicultural engagement was represented by four parcel indicators, two reflecting Korean Cultural Orientation and two reflecting Chinese Cultural Orientation, consistent with a bidimensional view of acculturation in which heritage and receiving cultural orientations may coexist rather than represent opposite ends of a continuum.

Model fit was evaluated using χ^2^, CFI, TLI, RMSEA, and SRMR. Maternal educational attainment and Korean-language proficiency were included as covariates because previous studies have identified these factors as relevant to marriage migrant women’s educational participation, parenting stress, and parenting efficacy (Hong & Shin, 2015; Seo, 2021). Preliminary analyses also indicated that both variables were significantly associated with parenting efficacy; therefore, they were retained as covariates in the structural model. Indirect effects were tested using bias-corrected bootstrapping with 2000 resamples, with statistical significance determined when the 95% confidence interval did not include zero.

The sample size was evaluated in relation to the complexity of the proposed model rather than solely by a fixed participant-to-parameter ratio. The model comprised four latent constructs represented by 15 indicators and was specified parsimoniously using theoretically defined subscales and parcels. Although the sample of 158 participants permitted estimation of the proposed model, it should be regarded as modest for SEM; accordingly, the stability and generalizability of the estimates are interpreted with appropriate caution. Maternal educational attainment and Korean-language proficiency were included as observed covariates and specified as direct predictors of parenting efficacy.

## 3. Results

### 3.1. Correlation of Main Variables

Pearson’s correlation analyses were conducted to examine the relationships among the study variables. Communication anxiety was positively associated with all three dimensions of parenting stress, including Parental Distress (r = 0.37, *p* < 0.001), Difficult Child (r = 0.35, *p* < 0.001), and Parent–Child Dysfunctional Interaction (r = 0.36, *p* < 0.001). In contrast, communication anxiety was negatively associated with several dimensions of parenting self-efficacy, including Discipline (r = −0.35, *p* < 0.001), Instrumental Care (r = −0.21, *p* < 0.05), and Teaching (r = −0.40, *p* < 0.001). Korean cultural orientation was negatively associated with communication anxiety (r = −0.30, *p* < 0.001) and with all three dimensions of parenting stress: Parental Distress (r = −0.42, *p* < 0.001), Difficult Child (r = −0.24, *p* < 0.01), and Parent–Child Dysfunctional Interaction (r = −0.20, *p* < 0.05). Korean cultural orientation was also positively associated with Nurturance (r = 0.22, *p* < 0.01), whereas its associations with the other dimensions of parenting self-efficacy were not statistically significant. In contrast, Chinese cultural orientation was positively associated with Korean cultural orientation (r = 0.33, *p* < 0.001) but was not significantly related to parenting stress or parenting self-efficacy.

All three dimensions of parenting stress were negatively associated with most dimensions of parenting self-efficacy. These correlations ranged from r = −0.26 to r = −0.75, with the strongest association observed between Parent–Child Dysfunctional Interaction and Discipline (r = −0.75, *p* < 0.001). Overall, the findings indicate that greater communication anxiety and lower Korean cultural orientation were associated with higher levels of parenting stress, whereas higher parenting stress was consistently associated with lower parenting self-efficacy. In contrast, Chinese cultural orientation showed little direct association with parenting stress or parenting self-efficacy. No substantial problems were identified regarding missing data, normality, outliers, or multicollinearity; therefore, maximum likelihood estimation was retained (Table 1).

### 3.2. Measurement Model

The measurement model was examined using confirmatory factor analysis. The model showed an overall marginal-to-acceptable fit to the data, χ^2^(84) = 149.00, *p* < 0.001, CFI = 0.92, TLI = 0.90, RMSEA = 0.07, and SRMR = 0.06. Although the RMSEA and SRMR indicated acceptable fit, the CFI and particularly the TLI were close to conventional lower-bound criteria; the model fit was therefore interpreted cautiously. An alternative correlated two-factor specification treating Korean and Chinese cultural orientations as separate factors showed only slightly better fit than the original model, χ^2^(80) = 141.00, CFI = 0.93, TLI = 0.90, RMSEA = 0.07, and SRMR = 0.05; therefore, the theoretically specified bicultural-engagement factor was retained.

Communication anxiety was represented by three indicators, while bicultural engagement was represented by four parcel indicators reflecting Korean and Chinese cultural orientations. A one-factor CFA conducted prior to parceling showed acceptable fit for the 11 adapted items, χ^2^(44) = 72.36, *p* = 0.005, CFI = 0.96, TLI = 0.95, RMSEA = 0.06, and SRMR = 0.04. Parenting stress was indicated by Parental Distress, Difficult Child, and Parent–Child Dysfunctional Interaction, and parenting self-efficacy was represented by Discipline, Play, Nurturance, Instrumental Care, and Teaching. The indicators loaded adequately on their respective latent constructs, supporting the measurement structure. Convergent validity was evaluated using standardized factor loadings, composite reliability (CR), and average variance extracted (AVE), and discriminant validity was assessed by comparing the AVE of each construct with the squared correlations between constructs. CR values were acceptable across constructs, although the AVE for bicultural engagement was relatively low (0.45). The discriminant-validity criterion was satisfied across the constructs (Table 2).

**Figure 1:**
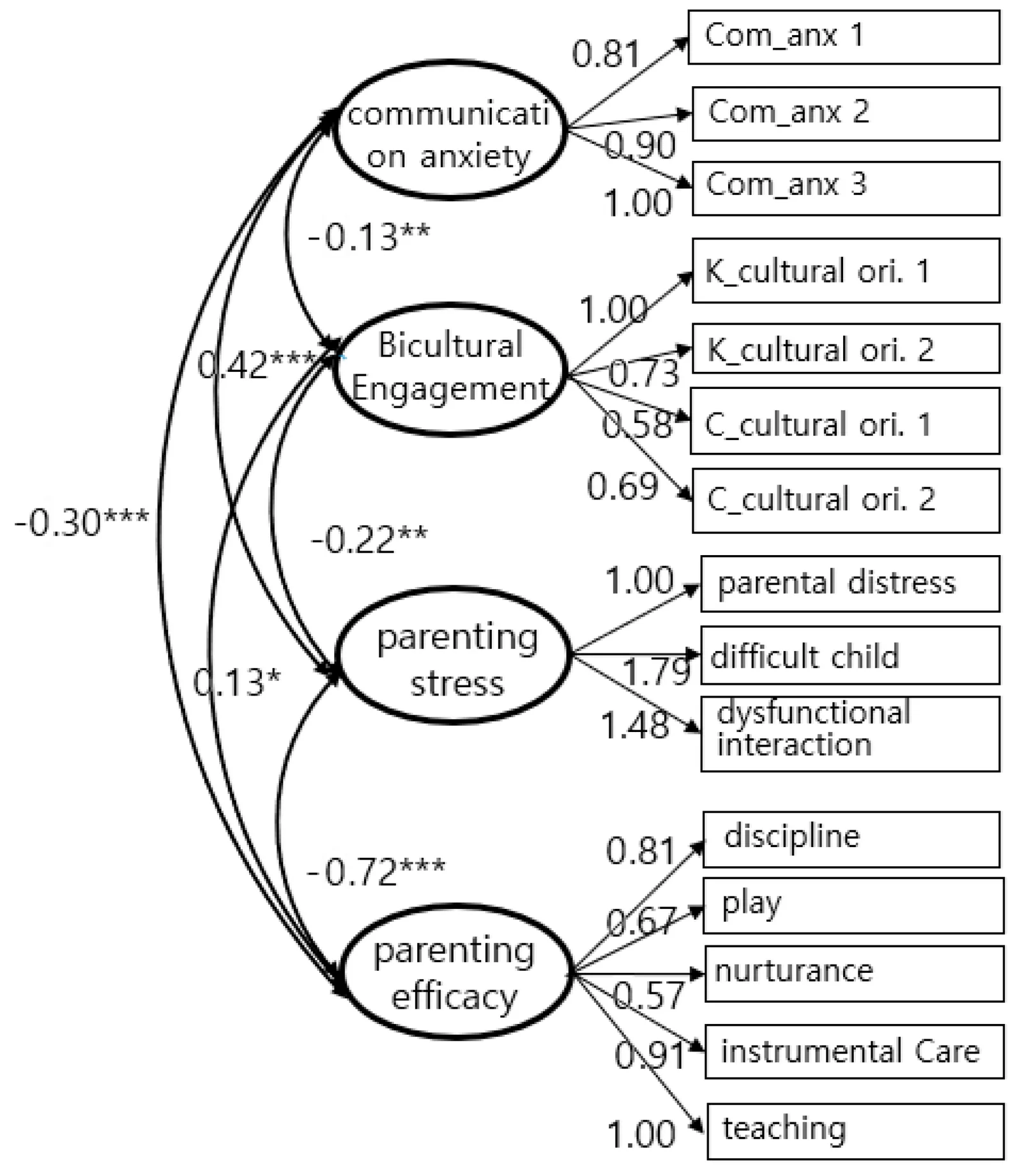
Measurement Model. * *p* < 0.05, ** *p* < 0.01, *** *p* < 0.001.

The latent constructs were significantly associated with one another. Communication anxiety was negatively associated with bicultural engagement and parenting efficacy, and positively associated with parenting stress. Bicultural engagement was negatively associated with parenting stress and positively associated with parenting self-efficacy. Parenting stress was negatively associated with parenting efficacy. These findings indicate that greater communication anxiety was associated with higher parenting stress and lower parenting self-efficacy, whereas stronger bicultural engagement was associated with lower parenting stress and higher parenting self-efficacy (Figure 1).

### 3.3. Structural Model and Mediation Effects

**Figure 2:**
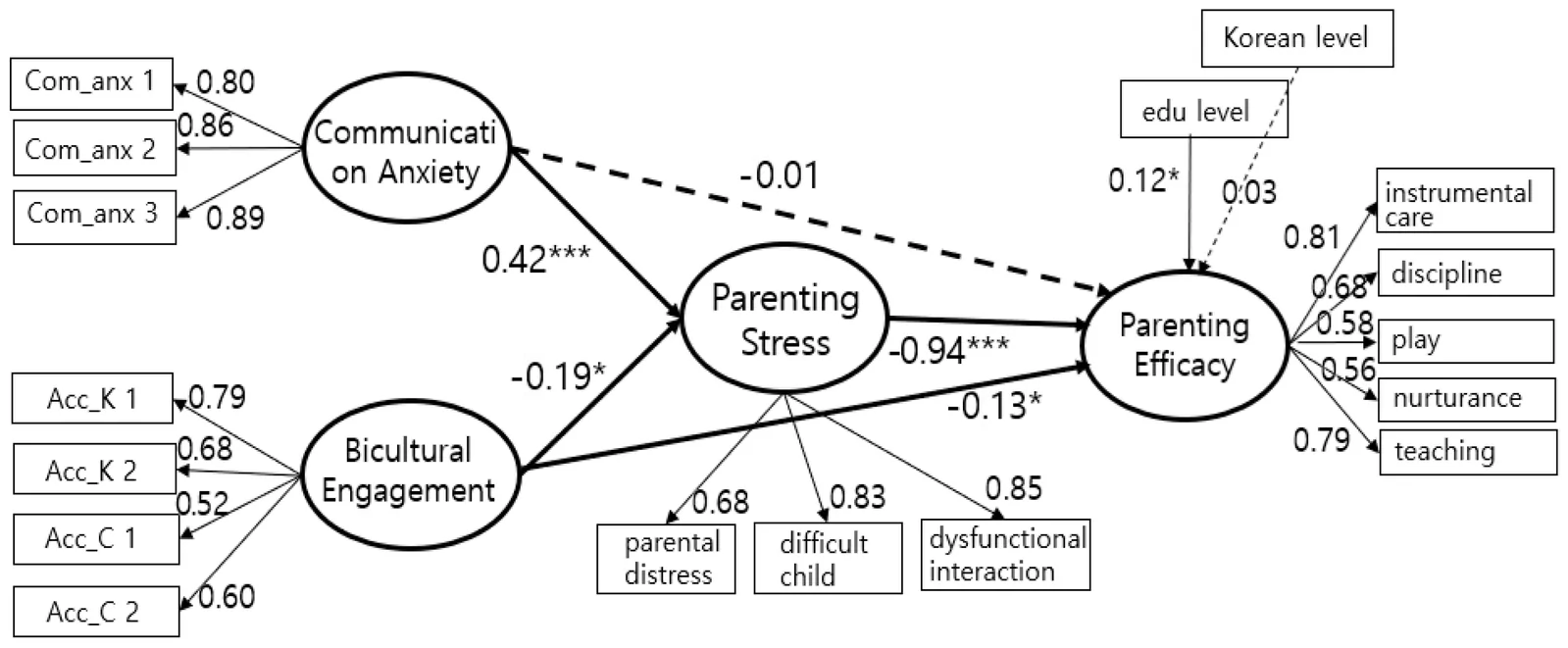
Final Model. * *p* < 0.05, *** *p* < 0.001. Note. Standardized path coefficients are reported. The loading of all observed variables was significant at *p* < 0.001 level.

The model results, including standardized coefficients, are presented in Table 3 and Figure 2. The structural model showed an overall marginal-to-acceptable fit to the data, χ^2^(108) = 204.32, CFI = 0.91, TLI = 0.90, RMSEA = 0.07, and SRMR = 0.07. Thus, although the absolute fit indices were within commonly accepted ranges, the incremental fit indices were close to their conventional lower bounds. The structural model showed that communication anxiety was positively associated with parenting stress (β = 0.42, *p* < 0.001), whereas bicultural engagement was negatively associated with parenting stress (β = −0.19, *p* < 0.05). Together, communication anxiety and bicultural engagement explained 22% of the variance in parenting stress (SMC = 0.22). Parenting stress was strongly and negatively associated with parenting efficacy (β = −0.94, *p* < 0.001). After accounting for parenting stress, the direct path from communication anxiety to parenting efficacy was not statistically significant (β = −0.01). In contrast, the indirect association through parenting stress was significant and negative (β = −0.39, 95% bias-corrected CI [−0.45, −0.11]), with a total association of β = −0.40. Thus, communication anxiety was associated with lower parenting efficacy primarily through higher parenting stress, indicating an indirect-only mediation pattern.

Bicultural engagement showed a negative direct association with parenting efficacy (β = −0.13, *p* < 0.05), whereas its indirect association through parenting stress was significant and positive (β = 0.20, 95% bias-corrected CI [0.01, 0.54]). Given the relatively wide confidence interval and its lower bound close to zero, this indirect association should be interpreted cautiously. Specifically, stronger bicultural engagement was associated with lower parenting stress, which in turn was associated with higher parenting efficacy. Because the direct and indirect paths operated in opposite directions, the total association was relatively small (β = 0.07), indicating an inconsistent mediation pattern.

Overall, communication anxiety, bicultural engagement, and parenting stress explained 72% of the variance in parenting efficacy (SMC = 0.72). These findings identify parenting stress as an important intervening mechanism in the associations of communication anxiety and bicultural engagement with parenting efficacy among Chinese marriage migrant women in Korea.

## 4. Discussion

The findings showed two distinct pathways linking migration-related experiences with parenting efficacy. Communication anxiety was associated with higher parenting stress and showed a significant negative indirect association with parenting efficacy through parenting stress. Bicultural engagement, in contrast, was associated with lower parenting stress and showed a positive indirect association with parenting efficacy, although its direct association with parenting efficacy was negative. Together, these findings highlight parenting stress as an important intervening process while also indicating that communication anxiety and bicultural engagement operate differently in relation to parenting.

First, communication anxiety was positively associated with parenting stress, whereas bicultural engagement was negatively associated with parenting stress. Communication difficulties experienced by marriage migrant women may involve not only limited Korean-language proficiency but also concerns about misunderstanding, making mistakes, or being negatively evaluated during interactions (Ko & Kim, 2024). These concerns may become particularly stressful in parenting situations that require communication with teachers, healthcare providers, and other institutions (J. Kim et al., 2022; S. Lee & Lee, 2022). In contrast, bicultural engagement involves maintaining an orientation toward Chinese culture while also participating in Korean cultural and social contexts. For marriage migrant women who have established family lives in Korea, engagement with both cultures may provide resources for dealing with parenting demands and reduce uncertainty in everyday parenting.

Second, parenting stress mediated the relationship between communication anxiety and parenting efficacy, while the direct path was not significant. This finding suggests that communication anxiety may be related to parenting efficacy mainly when communication difficulties become part of the burden of parenting. Parenting efficacy reflects parents’ confidence in managing everyday parenting tasks (Back & Jang, 2023; Che et al., 2026; Y.-J. Jang & Yin, 2023). When parenting stress persists, however, parents may perceive everyday demands as more difficult to manage, which may in turn be related to lower confidence in their parenting role (H. J. Kim & Lim, 2022; Song et al., 2022). Thus, the association between communication anxiety and parenting efficacy appears to be closely linked to the level of stress experienced in parenting.

Third, the relationship between bicultural engagement and parenting efficacy showed a more complex pattern. Bicultural engagement was associated with lower parenting stress, which in turn was associated with higher parenting efficacy. However, its direct association with parenting efficacy was negative. One possible explanation is that engagement with both Korean and Chinese cultural contexts may provide access to a wider range of information and social resources while also exposing mothers to different expectations about parenting and education. These different standards may affect how mothers evaluate their own parenting. Thus, bicultural engagement may help reduce some of the stress involved in parenting without necessarily leading directly to higher parenting efficacy. Given the cross-sectional design of this study, this interpretation should be regarded as tentative. Longitudinal research could clarify the temporal ordering of bicultural engagement, parenting stress, and parenting efficacy, while qualitative studies could examine how mothers negotiate potentially differing parenting expectations across Chinese and Korean cultural contexts.

Fourth, communication anxiety and bicultural engagement were differently associated with parenting. Communication anxiety refers to anxiety experienced in specific interpersonal situations, whereas bicultural engagement concerns involvement with both heritage and receiving cultural contexts. A mother may be engaged with both Korean and Chinese cultures while still feeling anxious when communicating in Korean in particular situations. Likewise, confidence in Korean communication does not necessarily imply weaker ties to Chinese culture. These findings suggest that communication-related experiences and bicultural engagement represent different aspects of the experiences of marriage migrant women and should therefore be considered separately when examining parenting adaptation.

The present findings also appear to share some features with research conducted among immigrant mothers in other national and cultural contexts. A systematic review of parenting self-efficacy in immigrant families found that parents’ confidence in their parenting role is closely intertwined with acculturation-related stress and adjustment across diverse immigrant populations (Boruszak-Kiziukiewicz & Kmita, 2020). Similarly, among Mexican-origin Latina immigrant mothers in the United States, immigration-related stress was associated with parenting stress and, through parenting stress and emotional support, with less positive parenting practices (Fuentes-Balderrama et al., 2023). Research involving Chinese immigrant mothers in Canada has likewise shown that stress, social support, and cultural orientations are related to parenting practices (Su & Hynie, 2011). Taken together, these studies suggest that the broader process identified here—the intersection of migration-related demands, parenting stress, and parental functioning—may extend beyond Chinese marriage migrant women in Korea. At the same time, the specific forms of communication anxiety and bicultural engagement examined in this study are embedded in the Chinese–Korean context, including the need to communicate in Korean and to negotiate parenting expectations across Chinese and Korean cultural settings. The present findings should therefore be viewed as potentially transferable at the level of the underlying process rather than directly generalizable in magnitude or form to migrant mothers in other sociocultural contexts.

The findings provide direct support for considering communication anxiety and parenting stress as relevant correlates of parenting efficacy among Chinese marriage migrant women. The more specific programmatic suggestions that follow extend beyond what was directly tested in the present study and should therefore be regarded as directions for practice and future intervention research rather than as empirically established intervention effects. Previous research reported that host-country language skills were closely related to migrant women’s adaptation and participation in social activities (Tarakcioglu & Ciceklioglu, 2022). Role-play and situational practice involving parent–teacher meetings, healthcare visits, administrative procedures, and parent gatherings may therefore be useful. Such activities may help women become more comfortable using Korean in situations directly related to their children.

Second, support for marriage migrant women may benefit from taking bicultural engagement into account. Participation in Korean society does not require distancing from one’s heritage culture. Parent education, self-help groups, and shared childcare activities can provide opportunities to exchange parenting information while maintaining cultural ties. Programs that include both women from the same country of origin and Korean parents may also help expand social networks and reduce isolation. Couple education and counseling may be useful when differences in cultural expectations affect family roles or parenting.

Third, the findings suggest potential value in addressing parenting stress more directly. Counseling, parenting coaching, and stress management programs may help reduce parenting burden, but support should not focus only on mothers. Spouses and other family members may also need to be involved because parenting stress is often related to the division of family responsibilities and the quality of family relationships. Programs that include cognitive and emotional coping strategies, such as mindful parenting programs (Tan et al., 2024), may also be helpful.

Fourth, early childhood education settings can play an important role in reducing communication-related difficulties. Parent–teacher communication is necessary for sharing information about children, but it can also be stressful for parents who are not confident in Korean. Providing notices in plain Korean, multilingual summaries of important information, and simple guides for common consultation situations may make communication easier. These supports could be introduced first in institutions serving relatively large numbers of children from multicultural families and expanded based on their usefulness.

Several limitations should be considered. First, parenting experiences may differ depending on access to support services and social support (J. S. Kim, 2018). Because the participants in this study were recruited from Seoul, Gyeonggi Province, and Incheon, the findings may not fully reflect the experiences of marriage migrant women living in smaller cities or rural areas. Future studies should include participants from a wider range of regions and cultural backgrounds. Second, all variables were measured through self-report questionnaires, raising the possibility of common method bias and socially desirable responding. Future research could include interviews, observations, or reports from spouses and teachers. Third, the present model did not include other factors that may be relevant to parenting, such as spousal support, division of family roles, social networks, economic conditions, discrimination, length of residence, and child characteristics. Including these variables may provide a fuller understanding of parenting among marriage migrant women. Fourth, the cross-sectional and correlational design precludes conclusions about temporal or causal relationships among communication anxiety, bicultural engagement, parenting stress, and parenting efficacy. Longitudinal or cross-lagged designs are needed to determine the direction and possible reciprocal nature of these associations.

Despite these limitations, this study contributes to the literature by distinguishing communication anxiety from bicultural engagement and by examining parenting stress as a mediator of their relationships with parenting efficacy. The findings suggest that the parenting experiences of Chinese marriage migrant women cannot be understood only in terms of Korean-language proficiency or adjustment to Korean culture. Their continued engagement with both Korean and Chinese cultural contexts, anxiety in everyday communication, and stress related to parenting should also be considered. These findings may inform Korean-language education, parent education, counseling, and family support for marriage migrant women.

## 5. Conclusions

In conclusion, this study suggests that the parenting experiences of Chinese marriage migrant women in Korea are related not only to communication anxiety and bicultural engagement, but also to the stress they experience in carrying out their parenting roles. Parenting stress appears to be particularly important in understanding how these migration-related experiences are associated with parenting efficacy. With respect to the two research questions, communication anxiety showed a significant indirect association with parenting efficacy through parenting stress, whereas bicultural engagement showed opposing direct and indirect associations with parenting efficacy. The findings therefore suggest that support for marriage migrant women should address communication difficulties and parenting stress while also recognizing their continued engagement with both Korean and Chinese cultural contexts. This may provide a useful basis for developing more appropriate language, parenting, and family support programs for Chinese marriage migrant women in Korea. Because the participants were recruited from Seoul, Gyeonggi Province, and Incheon, however, the findings should be generalized to Chinese marriage migrant women in other regions with caution.

## Figures and Tables

**Table 1:** Descriptive Statistics and Correlations among the Main Variables.

	1	2	3	4	5	6	7	8	9	10	11
1	1										
2	−0.30 ***	1									
3	0.09	0.33 ***	1								
4	0.37 ***	−0.42 ***	−0.07	1							
5	0.35 ***	−0.24 **	0.01	0.50 ***	1						
6	0.36 ***	−0.20 *	0.01	0.55 ***	0.70 ***	1					
7	−0.35 ***	0.12	−0.01	−0.48 ***	−0.66 ***	−0.75 ***	1				
8	−0.13	0.14	0.05	−0.36 ***	−0.55 ***	−0.45 ***	0.48 ***	1			
9	−0.10	0.22 **	0.04	−0.33 ***	−0.53 ***	−0.41 ***	0.50 ***	0.57 ***	1		
10	−0.21 *	0.06	0.01	−0.26 **	−0.33 ***	−0.42 ***	0.46 ***	0.35 ***	0.35 ***	1	
11	−0.40 ***	0.12	0.06	−0.39 ***	−0.54 ***	−0.56 ***	0.69 ***	0.47 ***	0.45 ***	0.53 ***	1
M	2.69	3.27	3.88	2.79	2.54	2.51	3.37	3.60	3.80	3.38	3.26
SD	0.96	0.65	0.62	0.55	0.74	0.64	0.85	0.81	0.68	0.76	0.88
skewness	−0.79	0.14	−0.97	−0.53	−0.46	−0.27	−0.45	−0.58	−0.74	−0.41	−0.40
kurtosis	0.11	0.37	−0.02	−0.12	0.23	−0.24	−0.08	−0.09	−0.07	0.07	−0.05

* *p* < 0.05, ** *p* < 0.01, *** *p* < 0.001; 1. communication anxiety, 2. Korean cultural orientation, 3. China cultural orientation, 4. parenting stress_parental distress, 5. parenting stress_difficult child, 6. parenting stress_dysfunctional interaction, 7. parenting efficacy_discipline, 8. parenting efficacy_play, 9. parenting efficacy_nurturance, 10. parenting efficacy_instrumental Care, 11. parenting efficacy_teaching.

**Table 2:** Measurement Properties of the Latent Constructs and Indicators.

Construct	Indicator	M (SD)	α	Std. Loading (λ)	CR	AVE
Communication Anxiety	Parcel 1	2.69 (0.96)	0.92	0.84	0.91	0.77
	Parcel 2			0.91		
	Parcel 3			0.88		
Bicultural Engagement	Kor. Orientation Parcel 1	3.27 (0.65)	0.83	0.78	0.76	0.45
	Kor. Orientation Parcel 2			0.71		
	Chi. Orientation Parcel 1	3.88 (0.62)	0.83	0.57		
	Chi. Orientation Parcel 2			0.60		
Parenting Stress	Parental Distress	2.79 (0.55)	0.71	0.79	0.89	0.72
	Difficult Child	2.54 (0.74)	0.87	0.87		
	Dysfunctional Interaction	2.51 (0.64)	0.82	0.89		
Parenting Efficacy	Discipline	3.37 (0.85)	0.85	0.83	0.88	0.59
	Play	3.60 (0.81)	0.86	0.74		
	Nurturance	3.80 (0.68)	0.79	0.74		
	Instrumental Care	3.38 (0.76)	0.78	0.69		
	Teaching	3.26 (0.88)	0.80	0.83		

**Table 3:** Mediation Analysis of Final Model.

Path	Direct Effect (β)	Indirect Effect (β)	Total Effect (β)	SMC	
Communication Anxiety ⇒ Parenting Stress	0.42 ***		0.42	0.22	
Communication Anxiety ⇒ Parenting Efficacy	−0.01	−0.39	−0.40	0.72	
Bicultural Engagement ⇒ Parenting Stress	−0.19 *		−0.19		
Bicultural Engagement ⇒ Parenting Efficacy	−0.13 *	0.20	0.07		
path	B	S. E.	β	Bias-corrected 95% CI for β	
				lower	upper
Communication Anxiety ⇒ Parenting Stress ⇒ Parenting Efficacy	−0.17	0.07	−0.39 *	−0.45	−0.11
Bicultural Engagement ⇒ Parenting Stress ⇒ Parenting Efficacy	0.11	0.14	0.20 *	0.01	0.54

Note. SMC = squared multiple correlation. * *p* < 0.05, *** *p* < 0.001.

## Data Availability

The data presented in this study are available from the corresponding author upon reasonable request. The data are not publicly available due to privacy and confidentiality restrictions.
